# Increased levels of palmitoylethanolamide and other bioactive lipid mediators and enhanced local mast cell proliferation in canine atopic dermatitis

**DOI:** 10.1186/1746-6148-10-21

**Published:** 2014-01-14

**Authors:** Francesca Abramo, Luca Campora, Francesco Albanese, Maria Federica della Valle, Luigia Cristino, Stefania Petrosino, Vincenzo Di Marzo, Vincenzo Miragliotta

**Affiliations:** 1 Department of Veterinary Science, University of Pisa, Viale delle Piagge 2, Pisa, 56124, Italy; 2 Clinica Veterinaria S. Clemente, 7, Via Pietro Benvenuti, Arezzo, 52100, Italy; 3 Institute of Biomolecular Chemistry, Consiglio Nazionale delle Ricerche, Pozzuoli, Naples, Italy; 4 Science Information and Documentation Centre (CeDIS), Innovet Italia Srl, Milano, Italy

**Keywords:** Atopic dermatitis, Dog, Acylethanolamides, Palmitoylethanolamide, Mast cells

## Abstract

**Background:**

Despite the precise pathogenesis of atopic dermatitis (AD) is unknown, an immune dysregulation that causes Th2-predominant inflammation and an intrinsic defect in skin barrier function are currently the two major hypotheses, according to the so-called outside-inside-outside model. Mast cells (MCs) are involved in AD both by releasing Th2 polarizing cytokines and generating pruritus symptoms through release of histamine and tryptase. A link between MCs and skin barrier defects was recently uncovered, with histamine being found to profoundly contribute to the skin barrier defects.

Palmitoylethanolamide and related lipid mediators are endogenous bioactive compounds, considered to play a protective homeostatic role in many tissues: evidence collected so far shows that the anti-inflammatory effect of palmitoylethanolamide depends on the down-modulation of MC degranulation.

Based on this background, the purpose of the present study was twofold: (a) to determine if the endogenous levels of palmitoylethanolamide and other bioactive lipid mediators are changed in the skin of AD dogs compared to healthy animals; (b) to examine if MC number is increased in the skin of AD dogs and, if so, whether it depends on MC in-situ proliferation.

**Results:**

The amount of lipid extract expressed as percent of biopsy tissue weight was significantly reduced in AD skin while the levels of all analyzed bioactive lipid mediators were significantly elevated, with palmitoylethanolamide showing the highest increase.

In dogs with AD, the number of MCs was significantly increased in both the subepidermal and the perifollicular compartments and their granule content was significantly decreased in the latter. Also, in situ proliferation of MCs was documented.

**Conclusions:**

The levels of palmitoylethanolamide and other bioactive lipid mediators were shown to increase in AD skin compared to healthy samples, leading to the hypothesis that they may be part of the body’s innate mechanisms to maintain cellular homeostasis when faced with AD-related inflammation. In particular, the increase may be considered a temptative response to down-regulating the observed elevation in the number, functionality and proliferative state of MCs in the skin of AD dogs. Further studies are warranted to confirm the hypothesis.

## Background

Atopic dermatitis (AD) is an itchy chronic skin disease frequently observed in dogs. Although not completely understood, the aetiology of the disease is multifactorial and depends on complex interactions between genetic and environmental factors. An immune dysregulation that causes Th2-predominant inflammation and an intrinsic defect in skin barrier function are currently the two major hypotheses, both for human and canine AD [1-3]. Strong debate is still ongoing on whether changes observed in skin barrier function are the cause or consequence of AD inflammatory changes [4].

It is generally assumed that skin mast cells (MCs) contribute to AD inflammation [1,5], as recently supported by two outstanding scientific papers [6,7]. MCs are immune-inflammatory tissue-localized cells and although they reside in almost all of the major organs and tissues of the body, they are strategically located at host/environment interfaces, such as the skin, the gastrointestinal and urogenital tracts, and the airways. Yet, they are present also in many other tissues, like the heart, brain, kidneys and joints [8,9]. Once activated by either IgE-dependent or IgE-independent, MCs degranulate, a process that results in the exocytosis of an impressive array of pro-inflammatory and immuno-regulatory mediators that initiate immediate phase inflammatory responses and late phase reactions [10,11]. Particularly, MCs release Th2 polarizing cytokines such as IL4, IL10, and IL13 which can induce a stimulated naive CD4+ T cell to become a Th2 cell when activated in the lymph node [12]. Moreover, they contribute to AD development by the generation of pruritus symptoms through release of histamine and tryptase [13-17]. MCs are bone marrow-derived cells that mature to become tissue-resident MCs after localizing in appropriate tissues. Stem cell factor (SCF), a fibroblast/keratinocyte derived chemokine (also known as c-kit or CD117 ligand) has been shown to be necessary for the maturation and proliferation of tissue-resident MCs, and to increase in both lesional and nonlesional skin of humans and dogs with AD [18]. Proliferation of skin MCs has been documented in human patients with AD [19], and MCs dispersed from adult human skin have been shown to proliferate under appropriate conditions [20]. Albeit an increase in MC number has been occasionally documented in canine AD [21,22], a study performed in healthy and AD dogs failed to confirm MC proliferation in the skin [23]. Moreover, another study reported MC density to be significantly lower in the lesional and nonlesional skin of AD dogs than in the skin of control animals [24].

Skin barrier dysfunction in canine AD has recently been explored. It mainly consist of abnormal stratum corneum ultrastructure and decreased ceramides, i.e., a family of lipid molecules, composed of sphingosine and a fatty acid [25-29]. Surprisingly, a link between MCs and skin barrier defects was recently uncovered, with histamine – the best known MC mediator - being found to decrease the expression of tight junction proteins, leading to the formation of a defective skin barrier [30].

N-acylethanolamines (NAEs) and 2-arachidonoyl-glycerol (2-AG) are bioactive derivatives of fatty acids, which have been identified in almost all mammalian tissues and body fluids [31-33]. Upon the identification of a particular NAE, i.e., N-arachidonoyl-ethanolamine (anandamide, AEA) as the endogenous ligand for the cannabinoid receptors (CBs) in the early nineties, interest in these bioactive lipid compounds dramatically increased [34]. N-palmitoyl-ethanolamine (PEA), a shorter and fully saturated AEA analogue, was also paid much attention in view of its general anti-inflammatory, pain relieving and neuroprotective properties [32,35,36]. Endogenous PEA as well as other NAEs are considered to play a protective homeostatic role in many tissues, including the skin [32,37].

Evidence collected so far shows that the broad biopharmacological effects of PEA mainly depend on the down-modulation of MC degranulation [38-40].

Based on this background, the purpose of the present study was twofold: (a) to determine if the endogenous levels of PEA and other bioactive lipid mediators are changed in the skin of AD dogs compared to healthy animals; (b) to examine if MC number is increased in the skin of AD dogs and, if so, whether it depends on MC in-situ proliferation.

## Methods

### Animals

In a prospective study, 5 healthy dogs (1 male and 4 females; age range, 4 to 13 years) and 5 dogs with AD (2 males and 3 females; age range, 1 to 11 years) were included in the study. Healthy dogs were client-owned dogs admitted for neutering. Dogs with AD were client-owned dogs referred for dermatologic problems; six or more of the criteria provided by Favrot et al. [41] were fulfilled for each of these subjects. Dogs with AD had clinical signs of erythema, alopecia, pruritus, and mild crusting of skin and had positive results for intradermal allergen testing for both outdoor and indoor environmental allergens, which supported the clinical diagnosis of AD. Other pruritic diseases, such as flea allergy dermatitis, adverse food reactions, sarcoptic mange, Malassezia dermatitis and superficial bacterial infections were excluded. In particular, all AD dogs underwent flea control measures and were fed a hypoallergenic restriction diet for 8 weeks prior to collection of skin samples to exclude allergies to fleas or food as possible causes of AD. When needed, dogs were treated for secondary yeast or bacterial infections of skin. Immediately before collection of skin samples, cytological examination of skin at planned collection sites was performed for each dog with AD; bacteria and fungi were not detected. Treatments for secondary bacterial or yeast infections were discontinued at least 2 to 3 weeks prior to collection of skin samples in these dogs. To avoid bias attributable to inclusion of skin samples with AD lesions from various anatomic sites, only samples of skin lesions from the ventral aspect of cervical region and axillae of dogs were obtained.

Samples collected from the abovementioned dogs served for total lipid and NAEs and 2-AG quantification, morphometrical and densitometrical assessment of MCs. Evaluation of MCs proliferation index (Pi) was performed in dogs with AD only.

Institutional ethical committee approval was not required for the study because AD skin samples were obtained and histologically examined for diagnostic purposes. Written informed consent was obtained from owners of all dogs for inclusion of their dogs in the study.

Ten dogs with previously diagnosed AD were included in the study for MC Pi assessment only, to obtain statistically reliable data: paraffin embedded skin samples were selected from the dermatohistopatholology archive of our institution. The diagnosis of AD was retrospectively re-evaluated and based on the presence of six or more of the set of criteria proposed by Favrot [41]. It was known that the dogs, when needed, had been treated for yeasts and/or bacterial secondary infections and that neither bacteria nor fungi were histological detected.

### Collection of tissue samples

Skin samples were obtained from the abdominal region of each of the 5 healthy dogs during deep anesthesia (isoflurane) for surgery (i.e., neutering) and from each of the 5 client-owned dogs with AD. These latter dogs were sedated with butorphanol tartrate and medetomidine hydrochloride for intradermal allergen testing; skin samples were collected by use of local anesthesia with lidocaine (2%).

Two 6-mm punch biopsies were collected from adjacent sites: neutering area in healthy dogs and lesional areas in AD subjects. One biopsy was snap-frozen in liquid nitrogen then stored at -80° until performing lipid extraction. The second biopsy was promptly immersed in a 10% neutral-buffered formalin solution (pH 7,4) and routinely processed for paraffin embedding. Five μm thick sections were stained with haematoxylin and eosin (H&E) and toluidin blue (TB) and examined via light microscopy. H&E stained sections were used for the general assessment of histological lesions while TB sections served for morphometrical and densitometical evaluation.

### Lipid extraction and quantification

Skin biopsies were homogenized in chloroform/methanol/Tris–HCl 50 mM pH 7.4 (2 : 1 : 1, v/v) containing 10 pmol of [2H]_8_-AEA, [2H]_4_-PEA and [2H]_4_-OEA, and 50 pmol of [2H]_5_-2-AG as internal deuterated standards (purchased from Cayman Chemicals, Ann Arbor, MI). The lipid-containing organic phase was dried down, weighed and prepurified by open-bed chromatography on silica gel. Fractions obtained by eluting the column with 9 : 1 (by vol) chloroform/methanol were analysed by liquid chromatography– atmospheric pressure chemical ionization–mass spectrometry (LC– APCI–MS) by using a Shimadzu HPLC apparatus (LC-10ADVP) coupled to a Shimadzu (LCMS-2010) quadrupole MS via a Shimadzu APCI interface. LC–APCI–MS analyses were carried out in the selected ion monitoring mode, using m/z values of 356 and 348 (molecular ions +1 for deuterated and undeuterated AEA), 304 and 300 (molecular ions +1 for deuterated and undeuterated PEA), 330 and 326 (molecular ions +1 for deuterated and undeuterated OEA), and 384.35 and 379.35 (molecular ions +1 for deuterated and undeuterated 2-AG). AEA, OEA, PEA and 2-AG concentrations were calculated by isotope dilution and are expressed as pmol per g of wet tissue weight. The concentrations of 2-AG were obtained by adding up to the amounts of the 2-isomer also those of the 1(3)-isomer, which mostly originates from the isomerization of the former during work-up.

### Morphometry and densitometry

**Figure 1:**
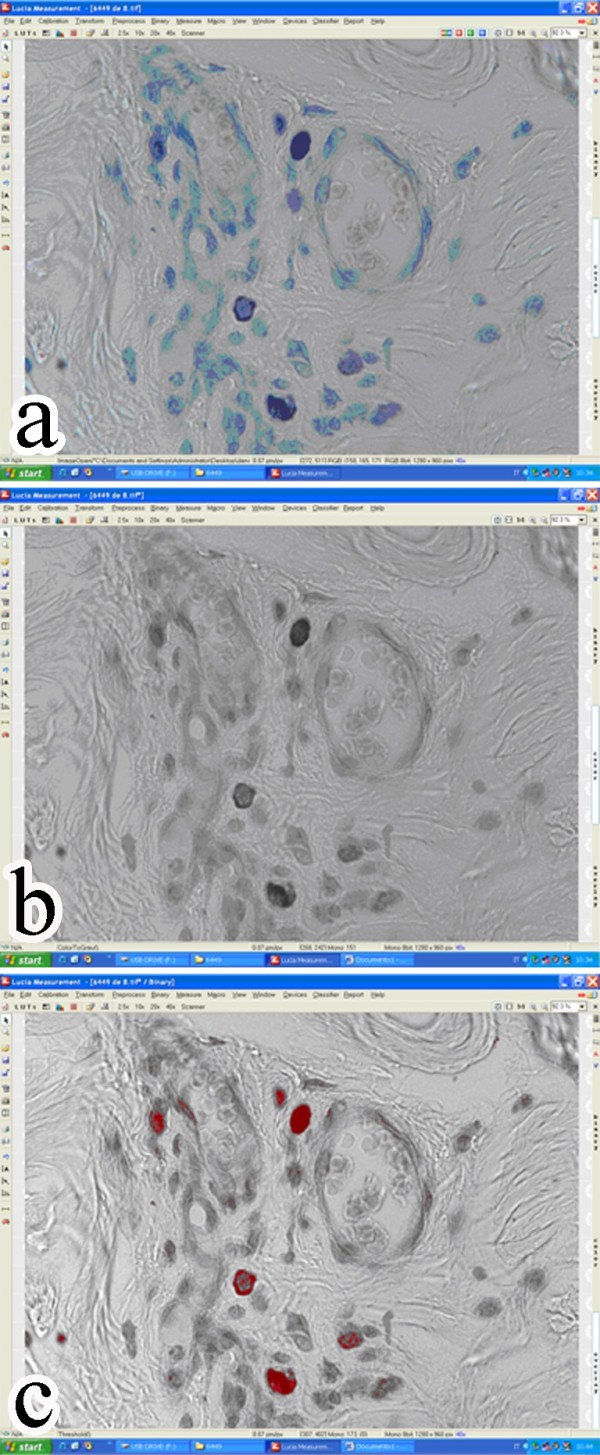
**Densitometry procedure. a)** Initial Toluidine blue stained captured image; **b)** binary (gray scale) image; **c)** red areas indicate measured granules.

The distribution of MCs and their granule content were assessed at high power field (magnification ×400) from the superficial (subepidermal) and perifollicular dermis. Toluidine blue cells were counted in 10 selected high power fields (HPFs) per section (400×). Values were expressed as number of counted cells per mm^2^. On the same selected HPFs cytoplasmic densitometry was assessed by applying the same pre-defined macro. Briefly, a binary image (gray levels) was obtained and granule content was measured as a mean gray value, as previously reported [38] (Figure 1). The Lucia (Nikon, Japan) analyser system was used.

### Immunohistochemistry

Superfrost-Plus mounted slides were used to detect proliferating MCs by double immunostaining with anti -cd117 and -Ki67 antibodies. Cd117 identifies the stem cell factor ligand receptor which is expressed on the cell membranes of both mature MCs and their earliest precursors. Ki67 is a cellular marker for proliferation present during all active phases of the cell cycle but absent from resting cells, the number of Ki67 labeled cells (nuclear immunoreactivity) on 100 cells (%) is worldwide accepted as the proliferation index (Pi). The immunohistochemical procedure was performed as follows. After deparaffinization and rehydrating, quenching of endogenous peroxidases was carried out by incubation with 3% H2O2 in distilled water (30 min, RT). Antigen unmasking was performed in citrate buffer pH 6 (UCS Diagnostic S.r.l., Morlupo, Rome, Italy) in microwave for 15 minutes. Non-specific binding was prevented by incubation of slides with a serum-free protein block (Super Block, Scy Tek Laboratories, Logan, UT, USA) (10 minutes, RT). Slides were subsequently incubated with the mouse monoclonal anti-Ki67 primary antibody (cat. M3060, Spring Bioscience, Pleasanton, CA, USA) 1:100 in PBS-Tween (PBT-1099 - UCS Diagnostic S.r.l., Morlupo, Rome, Italy) overnight at 4°C. The ULTRATEK-HRP kit (HS8-DAB Ultra Tek HRP, Scy Tek Laboratories, Logan, UT, USA) was used to visualise the immunoreaction. Glass slides were therefore rinsed in PBST for 10 minutes and incubated with a serum-free protein block (Super Block, Scy Tek Laboratories, Logan, Utah, U.S.A.) for 10 minutes. The rabbit polyclonal anti-CD117 antibody (cat. E1440, Spring Bioscience, Pleasanton, CA, USA) 1:400 in PBST (UCS Diagnostic S.r.l., Morlupo, Rome, Italy) was used (1 hour, RT) and the same ULTRATEK-HRP kit was used for secondary antibody and streptavidin-peroxidase complex. Immunoreactivity was visualised by a different chromogen (Vector® VIP, Vector Laboratories, Burlingame, CA, USA). Sections were then counterstained with Haematoxylin, dehydrated and mounted with a permanent mounting medium.

### Statistical analysis

The amounts of lipid extracts, NAEs and 2-AG were compared by the Wilcoxon signed-rank test. MCs counts and densitometry values were compared by the unequal variance t-test. For Pi mean percentage number of Ki67 immunolabelled MCs and standard deviation were calculated.

## Results

### Total lipid, NAE and 2-AG quantification

**Figure 2:**
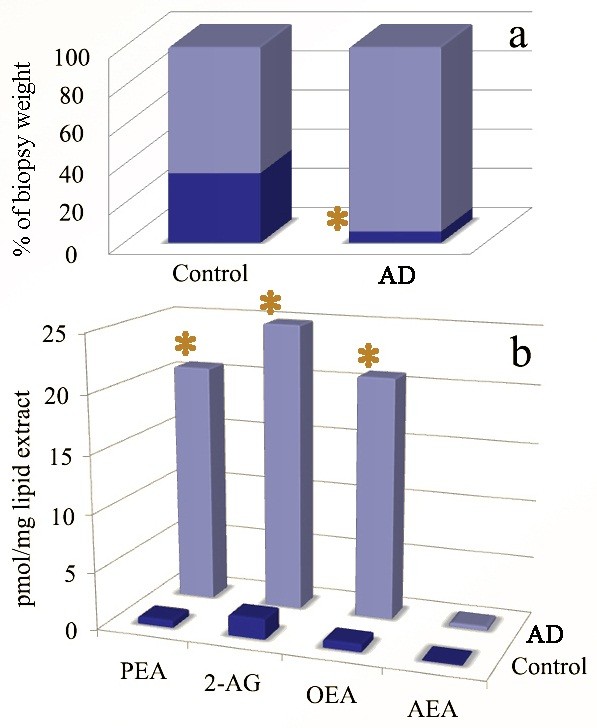
**Bar graphs showing lipid extract quantification. a)** lipid extract expressed as percent of biopsy tissue weight in control animals and dogs with atopic dermatitis; **b)** Quantification of different NAEs and 2-AG in control animals and dogs with atopic dermatitis. Asterisks indicate statistically significant differences (p < 0.05); AD = Atopic dermatitis.

The amount of lipid extract expressed as percent of biopsy tissue weight was significantly reduced (~6-fold) in the lesional skin of AD dogs, as compared to control dogs (35.63% vs 5.79% p < 0.05). Conversely, the levels of all analysed NAEs and 2-AG were significantly elevated (p < 0.05). In particular PEA levels showed the highest increase, being more than 30-fold higher in AD lesional skin than in normal non-atopic skin. OEA and 2-AG showed a 30- and 14-fold increase in AD lesional skin than in normal skin, respectively while AEA showed only a ~6-fold increase (Figure 2).

### Histological evaluation

**Figure 3:**
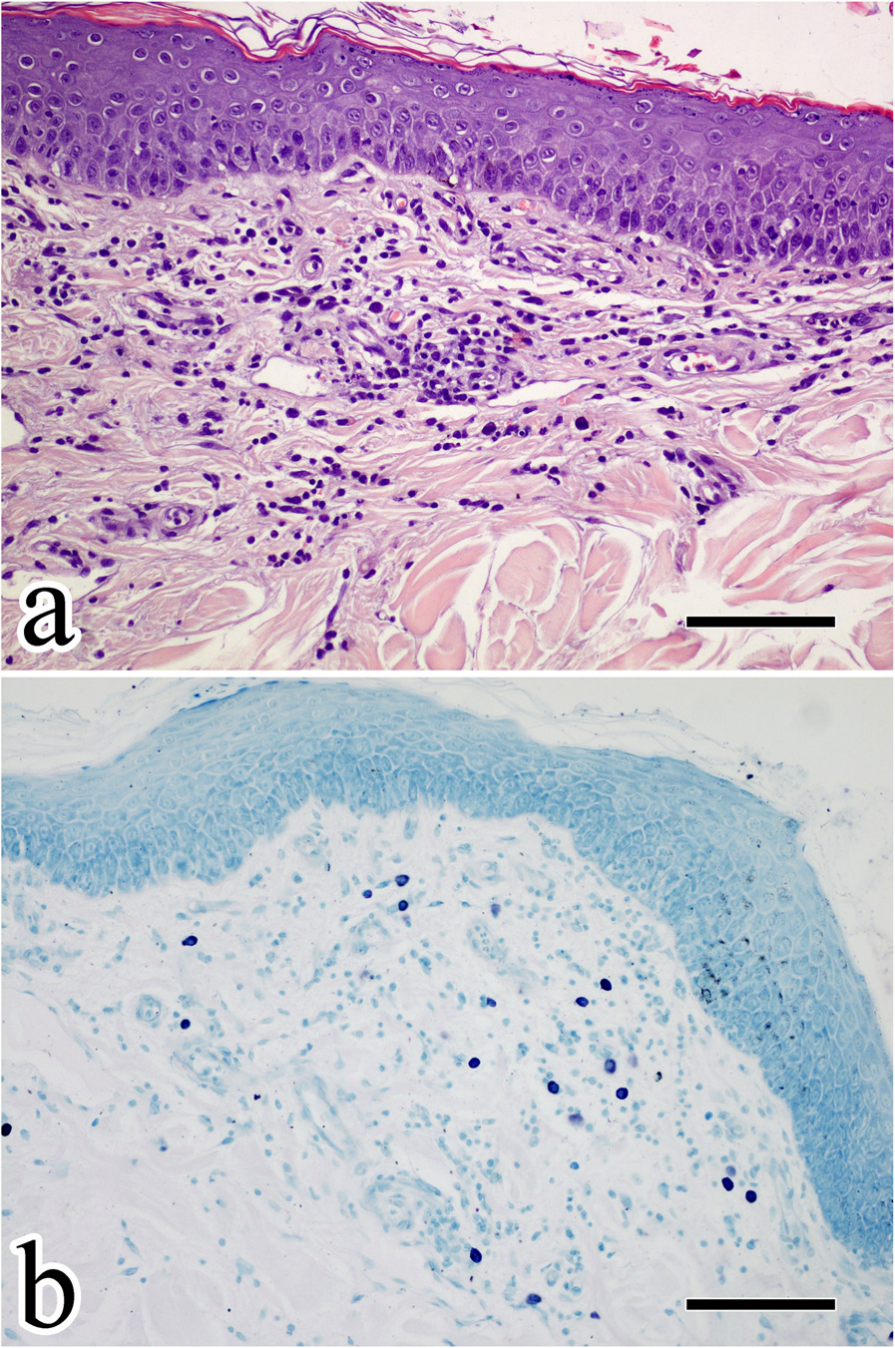
**Skin histopathology of dogs with AD. a)** Regular epidermal hyperplasia with mild spongiosis and superficial perivascular infitrate composed of lymphocytes, MCs and a few neutrophils; H&E stained section; **b)** Toluidine blue stained section showing methacromatic MCs infiltrating the superficial dermis. Scale bars = 100 μm.

Changes were not observed in skin of healthy dogs. Histopathological findings of specimens obtained from dogs with AD supported the clinical diagnosis. Briefly, a moderated epidermal hyperplasia with mild spongiosis was observed in all cases. Superficial dermal changes were related to a perivascular to interstitial infiltrate mainly composed by MCs, lymphocytes and few histiocytes and neutrophils. The superficial vascular plexus was prominent with reactive endothelial cells. Minimal subepidermal and perivascular oedema was observed (Figure 3).

### Mast cell morphometry and densitometry

**Figure 4:**
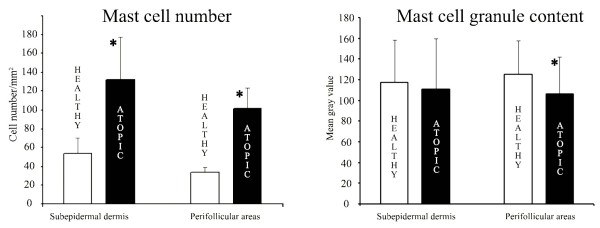
**Bar graphs showing MC count (left) and MC granule content (right) in control animals and dogs with atopic dermatitis.** Asterisks indicate statistically significant differences (p < 0.01).

Toluidine blue positive (metachromatic) MCs in normal skin were 53.90 ± 16.64 cells/mm^2^ and 34.34 ± 5.06 cells/mm^2^ in the subepidermal dermis and in the perifollicular areas, respectively. In the same selected areas, dogs with AD showed higher values (131.75 ± 45.69 cells/mm^2^ and 101.58 ± 21.84 cells/mm^2^). The difference was statistically significant for both sites (p < 0.01). In the perifollicular areas, a statistically significant decrease in MC granule content was observed in AD compared with normal canine skin (p < 0.01). Such a difference was not observed in the subepidermal compartment (Figure 4).

### Proliferation index of mast cells

**Figure 5:**
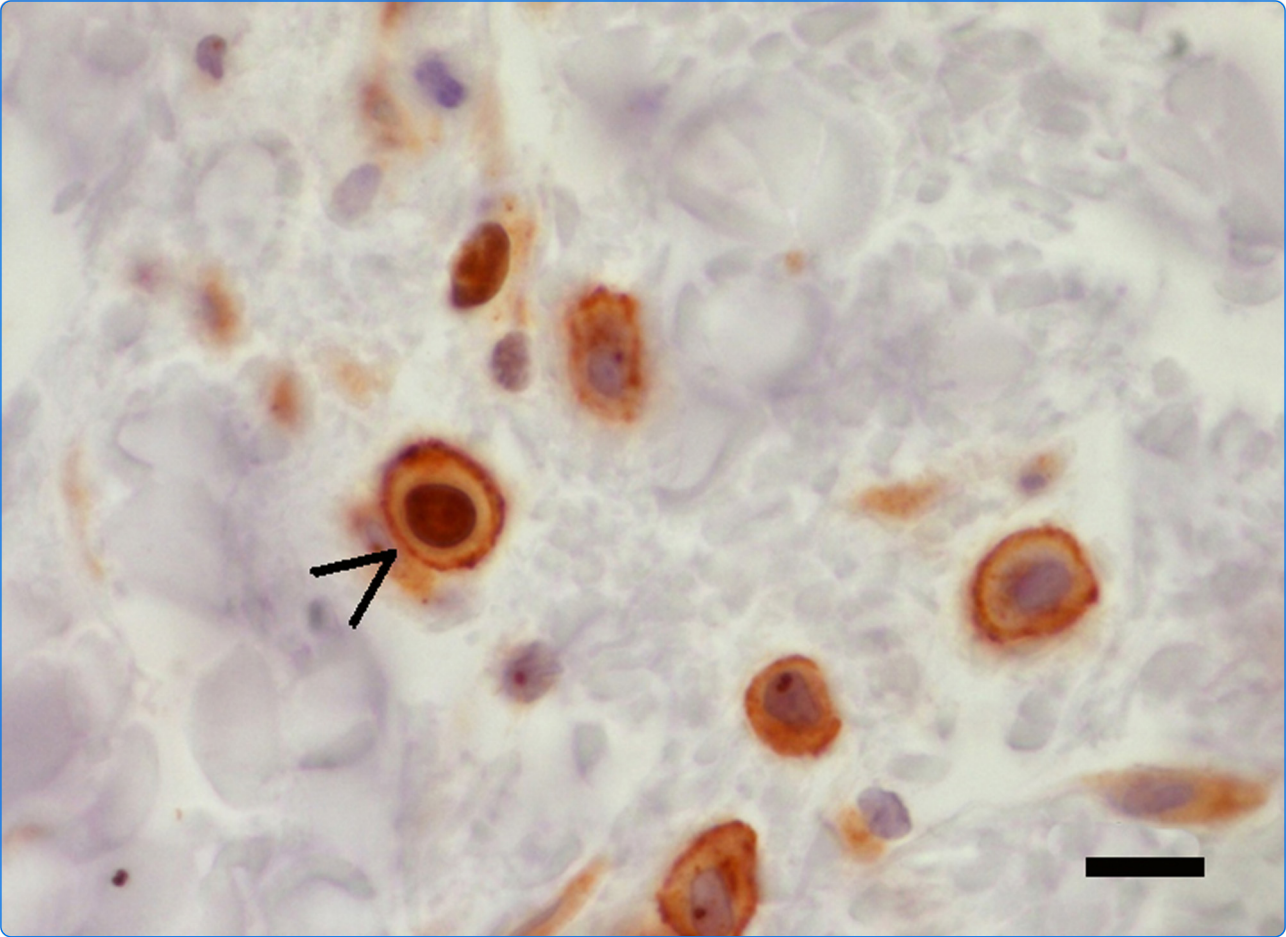
**Double immunohistochemistry with CD117 and Ki67.** Mast cells are CD117 positive membrane stained cells; One of them (arrowhead) shows also nuclear immunoreactivity (Ki67) indicating its proliferating status. Streptavidin-peroxidase method. Scale bar = 20 μm.

When the double immunostaining approach was performed, membrane CD117 immunoreactivity confirmed the presence of several MCs; some of them were also positive to the nuclear antigen Ki67, thus demonstrating their proliferating activity (Figure 5). Scattered individual or grouped keratinocytes in the basal layer of epidermis were also reactive to Ki67 and their proliferating status served as an internal positive control for the Ki67 reaction. Data analysis of the 5 dogs with AD prospectively selected showed a Pi mean value of 5.68 ± 2.25 while in the retrospectively included 10 dogs the mean value was 11.11 ± 6.47. When a cumulative analysis was performed, the obtained Pi value was 9.30 ± 5.95. MCs proliferation index values are reported in Table 1.

**Table 1:** MC proliferation index (Pi) data

**Group**	**Case No.**	**No. Areas**	**CD117 +**	**CD117 + / Ki67 +**	**Pi (%)**
1	1	16	56	4	7,14
	2	17	54	3	5,56
	3	18	80	6	7,50
	4	14	53	1	1,89
	5	23	158	10	6,33
				Group 1 mean	5,68
2	1	25	112	7	6,25
	2	15	46	6	13,04
	3	25	108	11	10,19
	4	11	37	4	10,81
	5	20	61	3	4,92
	6	20	51	12	23,53
	7	13	44	8	18,18
	8	20	104	15	14,42
	9	22	89	7	7,87
	11	25	106	2	1,89
				Group 2 mean	11,11
				**Cumulative mean**	**9,30**

Group 1 = prosective study. Group 2 = retrospective study.

## Discussion

The statistically significant decrease in total lipid content in the skin of dogs with AD showed in the present study fits with the documented skin barrier impairment in canine hypersensitivity skin disorders [3]. To the best of our knowledge, this is the first report showing the decrease in total skin lipid content in canine AD, previous studies having focused on particular composition defects (i.e., ceramides, free fatty acid, lamellar bodies) [42]. Particularly, abnormal stratum corneum ultrastructure and disorganized lipid lamellae were found in nonlesional and lesional skin of spontaneous and experimentally-induced canine AD [27,43-45]. Moreover, lower proportion of ceramides were found in both nonlesional and lesional skin of dogs with AD compared to that of normal dogs [20,24,39].

Opposite to the decrease of total lipid extracts, the levels of PEA and related lipid mediators (i.e., OEA and 2-AG) were shown here to increase in AD samples compared to healthy ones. Among all the analysed bioactive lipid compounds, PEA levels were more than 30-fold higher in AD lesional skin compared to normal non-atopic skin. Moreover, if one considers the total decrease of lipid content, the overall increase of PEA and the other examined lipid mediators is dramatic and once more might witness the role of NAEs and 2-AG in cellular homeostasis in the face of external stressors provoking, for example, inflammation. Currently, it is widely accepted that the endogenous formation of these bioactive lipid mediators plays a pro-homeostatic role, by being part of a protective response to cellular injury [32,46]. In particular, PEA levels have been measured in mammalian skin [47-50] and found to change in response to stressful conditions, both in animal models [51] and epidermal cell cultures [52]. The increase in tissue levels of PEA is now considered an auto-protective mechanism, while the decrease is usually regarded as an exhaustion of the response [53].

The hypothesis is highly substantiated by the favourable effects of PEA administration: mice with contact dermatitis [50], hypersensitive Beagle dogs [54] and cats with eosinophilic granuloma [38] all benefited from PEA treatment in terms of reduced allergic skin reactions.

Although the exact mechanism of PEA action is still a matter of debate, it is generally assumed that it depends on a multitarget pathway signalling, with the direct or indirect engagement of different receptors, such as CB, PPAR, TRPV1 and GPR55 receptor [32,34,37,53]. In line with this, we recently reported the immunolocalization of CB1 and CB2 in the skin of normal and AD dogs, the latter showing increased expression of these receptors [55]. Moreover, CB1 stimulation has been recently reported to limit excessive activation of hair follicle-associated MCs [56]. Compounds like PEA and OEA, which do not directly activate CB receptors, might instead produce anti-inflammatory actions via other targets such as PPARα and TRPV1 [57,58]. On a cellular level, the main target of PEA is considered to be the MC [32-34].

We found MC density to increase both in the subepidermal and the perifollicular compartment of atopic animals compared to healthy controls. Although it could be speculated that different sample locations (i.e., ventral neck in AD dogs versus abdominal region in healthy dogs) could partially explain the difference, one should consider that the distribution of MCs at various skin sites of dogs does not seem to differ among body regions, except for ears (pinnae) where MC number is significantly higher [59-61]. Moreover, our results are consistent with the previously shown increase in MC density in various skin sites of AD dogs compared to controls [22,62]. Nevertheless the overall scenario on the topic is still somewhat conflicting, MC numbers having been reported not to differ between healthy and AD canine skin [24]. A recent review [56] summarizes the main findings on MC number in human AD [63]: in acute lesions, MCs are normal in number but show degranulation [64]. In chronic lesions, however, their numbers are significantly increased, especially in areas of lymphocytic infiltration in the papillary dermis [64,65].

Our densitometric data suggest a different functionality of the two anatomic MC populations examined, since degranulation was found to be more prominent in perifollicular than subepidermal MCs. A specific MC population has been recently characterized in humans, harbouring the perifollicular connective tissue sheath, thus accounting for a possible different role of the two differently distributed MC populations [56]. A selective degranulating state of atopic skin MCs was reported in dogs by Welle and colleagues [24] who found a significantly lower MC density in both lesional and nonlesional skin samples of atopic dogs than in the skin of control dogs when stained for the two MC-specific proteases, i.e., tryptase and chymase. Moreover, our findings are consistent with those recently found by an electron microscopic study, showing that MCs from canine skin with AD exhibited piecemeal, anaphylactic and mixed pattern degranulation [22].

Given our morphometric data on the significantly increased MC count both in the subepidermal and perifollicular areas (i.e., almost a 3-fold higher value than in control skin), we asked if this could partly rely on in situ proliferation. We actually found Ki67-CD117 double positive cells, i.e. skin cells expressing the transmembrane tyrosine kinase growth factor receptor (c-kit or CD117 or SCF receptor) together with the proliferation nuclear marker Ki67. These are proliferating MCs, since CD117 immunoreactivity in canine skin clearly identify MCs [66]. These results support the hypothesis that during AD, accumulation of dermal MCs does not arise exclusively from recruitment of bone-marrow-derived precursor cells, as previously stated [23], but also partially from in situ proliferation. Although one cannot rule out the hypothesis that Ki67-labelled cells immature MCs which have just left the bloodstream to infiltrate the dermis and may be related to enhanced migration of MC precursors from the bloodstream as suggested by previous studies [23], the identification of in situ proliferation of resident MCs in the connective outer root sheath of follicles by Sugavara et al. [56] strongly support our hypothesis of in situ proliferation of resident MCs in canine AD.

## Conclusions

The current study adds new information on the so-called outside-inside-outside pathophysiological model of canine AD. At the skin barrier side (i.e., outside), our findings show a decreased of total lipid content. At the immune-inflammatory side of the disease (i.e. inside) we have shed new light on the body own “inflammatory dualism”, with an increased MC density and activation state and a concurrently increased production of the endogenous anti-inflammatory armamentarium (i.e., increased bioactive lipid compounds and especially PEA in the lesional skin of AD dogs compared to controls). Further studies are needed to deeply explore the eventual interplay among the changes reported here and to investigate whether PEA and related endocannabinoids play indeed an adaptive protective role in canine AD.

## Abbreviations

2-AG: 2-arachidonoyl-glycerol; AD: Atopic dermatitis; CBs: Cannabinoid receptors; FAAs: Fatty acid amides; H&E: Haematoxylin and eosin; HPFs: High power fields; IgE: Immunoglobulin-E; LC– APCI–MS: Liquid chrom.– atmosph. pressure chem. ionization–mass spectrometry; MCs: Mast cells; NAEs: N-acylethanolamines; AEA: N-arachidonoyl-ethanolamine, Anandamide; OEA: N-oleoyl-ethanolamine; PEA: N-palmitoyl-ethanolamine; Pi: Proliferation index; SCF: Stem cell factor; TB: Toluidin blue.

## Competing interests

Maria Federica della Valle is a scientific consultant for CeDIS (Science Information and Documentation Centre), Innovet Italia srl. None of the other authors declare a conflict of interest.

## Authors’ contributions

F Abramo, VM contributed to the conception and design of the study, data analysis and writing of the manuscript; L Campora was primarily involved in histological data production and acquisistion; F Albanese clinically selected the animals to be enrolled in the study; L Cristino and SP were primarily involved in biochemical data production and acquisistion; MFdV and VDM contributed to the final critical revision of the manuscript for their experience on the topic. All authors read and approved the final manuscript.
